# Targeting the parasite in the mosquito: rationale and practicality

**DOI:** 10.1186/1475-2875-11-S1-O5

**Published:** 2012-10-15

**Authors:** Robert E Sinden, MJ Delves, AM Blagborough

**Affiliations:** 1 Department of Life Sciences, Imperial College London, and the Jenner Institute, University of Oxford, UK

## 

From the very earliest days of malaria research and control it has been recognised that attacking the mosquito vectors is one of the most powerful interventions at our disposal. Recognising that these activities are, *sensu stricto*, attacking the population of parasites as they pass through the vectors, it is now appreciated that attacking the parasite *per se* within the mosquito vector has equal potential to contribute to malaria control, elimination or indeed eradication.

The rationale behind current anti-parasitic malaria transmission-blocking strategies is profound. Parasite populations in the mosquito host can be 10^–10^ smaller than those in the human host, offering reduced genetic/molecular potential with which the parasite can combat any intervention. Further, the parasites are, for the first 24 hours, extracellular and directly exposed to any intervention (drug; vaccine or other) delivered in the infectious bloodmeal. Finally, within the vector many of the accessible parasite and mosquito surface molecules have never been exposed to the human immune system, perhaps as a correlate to this they, by comparison with merozoite and sporozoite surface molecules, are neither antigenically variant, nor significantly polymorphic, thus offering stable and geographically widespread targets for intervention.

In response to the new calls for research to underpin possible elimination/eradication campaigns, current efforts are now describing potential and effective new drugs and vaccine strategies targeting the parasites in the mosquito bloodmeal. Recent studies examining the potency of transmission blocking vaccine candidates, and the identification of new and transmission blocking drugs will be reviewed. Questions must be asked as to what relevance the outputs of current laboratory based transmission blocking experiment have to the key population-based parameters that must be measured in the field. As an example:- If an intervention causes a 50% reduction in oocyst number in a laboratory population of mosquitoes, what impact, if any, will it have on the number of secondary cases in the field? Recent studies addressing these key questions will be reported.

